# An open-label randomized controlled trial of leflunomide in patients with acute SARS-CoV-2 omicron variant infection

**DOI:** 10.3389/fmed.2023.1218102

**Published:** 2023-07-18

**Authors:** Zhou Pan, Zhihui Wan, Yixuan Wang, Shiqian Zha, Jingyi Zhang, Hao Chen, Ke Hu

**Affiliations:** ^1^Department of Respiratory and Critical Care Medicine, Renmin Hospital of Wuhan University, Wuhan, China; ^2^East Campus, Renmin Hospital of Wuhan University, Wuhan, China

**Keywords:** leflunomide, coronavirus disease 2019 (COVID-19), omicron variant, sustained clinical recovery, viral shedding time

## Abstract

**Objective:**

To evaluate the efficacy and safety of leflunomide for the treatment of acute, symptomatic COVID-19.

**Methods:**

A single-center, open-label, randomized controlled trial was performed during an outbreak of SARS-CoV-2 Omicron variant in December 2022. Symptomatic patients within 5 days of COVID-19 onset were randomly allocated to receive 5 days of either symptomatic treatment with leflunomide or symptomatic treatment alone. The primary endpoint was time to sustained clinical recovery.

**Results:**

Fifty-seven participants were randomized into two groups: 27 received leflunomide plus symptomatic treatment and 30 were assigned to symptomatic treatment alone. Participants treated with leflunomide had a shorter fever duration [3.0 interquartile range (IQR, 2.0–4.0) days and 4.0 (IQR, 3.0–6.0) days, respectively (*p* = 0.027)] and reduced viral shedding [7 (IQR, 6–9.5) days and 9.0 (IQR, 7.5–12.0) days, respectively (*p* = 0.044)] compared with individuals treated with symptomatic treatment alone. However, there were no significant differences in time to sustained clinical recovery between the two groups [hazard ratio, 1.329 (95% confidence interval, 0.878–2.529); *p* = 0.207].

**Conclusion:**

In acute adult COVID-19 patients presenting within 5 days of symptom onset, leflunomide combined with symptomatic treatment reduced fever duration and viral shedding time.

**Clinical Trial Registration:**

https://www.chictr.org.cn/about.html, ChiCTR2100051684.

## Graphical abstract

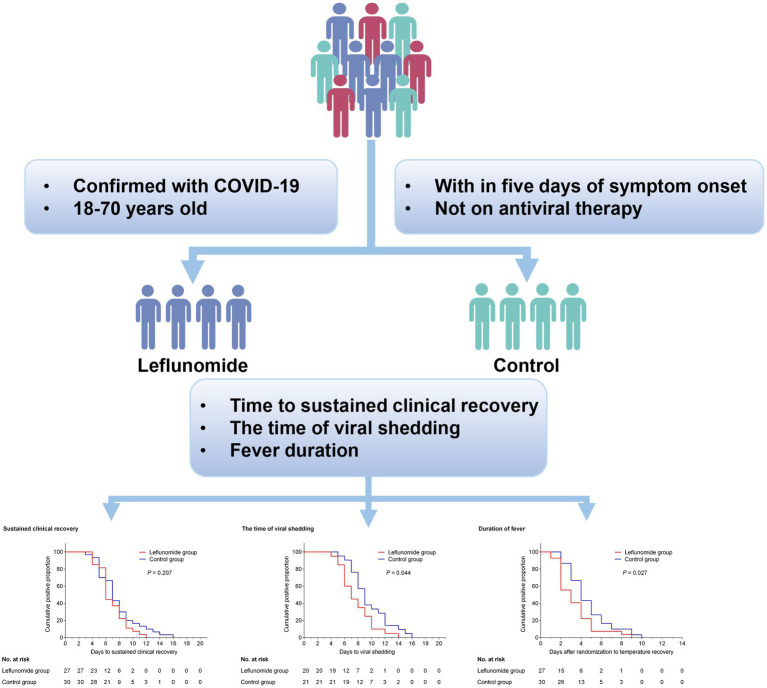

## Introduction

The emergence of the SARS-CoV-2 Omicron variant triggered a new wave of the COVID-19 pandemic in China in December 2022 and January 2023. Compared with the original SARS-CoV-2 strain, this variant has more mutations in the receptor-binding domain of the spike protein, significantly increasing its transmissibility and ability to evade the immune system (1). Therefore, safe and effective antiviral drugs are still needed to manage the clinical burden.

Nirmatrelvir/ritonavir, an oral antiviral drug, currently has an emergency use authorization from the U.S. Food and Drug Administration (FDA) for the treatment of mild-to-moderate COVID-19 outpatients at high risk of progression (2). In addition, azvudine, the nucleoside analog 2′-deoxy-2′-β-fluoro-4′-azidocytidine, reduces the time to first nucleic acid negative conversion in clinical trials (3–5). In August 2022, the Chinese National Health Commission granted the drug its license for the treatment of COVID-19 (6). However, the application of the two drugs was limited by their availability, price, and drug interactions. Novel, potent oral anti-SARS-CoV-2 medications are still required.

RNA virus replication, including that of SARS-CoV-2, depends on host *de novo* pyrimidine synthesis (7–9). Dihydroorotate dehydrogenase (DHODH) is the rate-limiting enzyme that catalyzes the fourth step of the pyrimidine biosynthesis pathway, and inhibiting pyrimidine synthesis in virus-infected cells effectively inhibits virus replication, as shown by researchers in Wuhan in the early phase of the pandemic in 2020 (10). Based on this study, we applied for an emergency small-scale interventional study of the DHODH inhibitor leflunomide and found that it may reduce the duration of viral shedding and length of hospitalization in patients with COVID-19 (11). However, our subsequent study did not show any advantage for leflunomide plus IFN-α-2a beyond IFN-α-2a alone in terms of duration of viral shedding in COVID-19 patients with prolonged post-symptomatic viral shedding (12). Given these results, we hypothesized that leflunomide is efficacious for patients with acute SARS-CoV-2 infection, and we tested this hypothesis by evaluating the efficacy and safety of leflunomide in a randomized controlled trial of patients with acute COVID-19.

## Methods

### Participants and ethics

This was an open-label, randomized controlled clinical trial. Sixty patients with a confirmed diagnosis of COVID-19 in the acute phase of infection within 5 days of onset were included. All participants were recruited from the Renmin Hospital of Wuhan University between December 8, 2022 and December 20, 2022 and were followed up to January 15, 2023. The Ethics Committee of Renmin Hospital of Wuhan University (WDRY2021-K122) reviewed and approved the study protocol, and the study was registered with the Chinese Clinical Trial Registry (ChiCTR2100051684, registered on September 30,2021; and refreshed on March 5, 2023). Written informed consent was obtained from each participant.

### Inclusion and exclusion criteria

The inclusion criteria were: (1) patients aged 18–70 years positive for SARS-CoV-2 by nucleic acid or antigen testing, meeting the Chinese diagnostic guidelines for COVID-19 (13), and with a total symptom score ≥ 3 (see Supplementary Table S1); (2) within 5 days of symptom onset; (3) provided written informed consent; (4) agreed to the collection of clinical samples; (5) if female and of reproductive age then not pregnant; (6) agreed to use effective contraception within 7 days of the last oral medication.

Candidates were excluded if: (1) they had severe vomiting or difficulty in taking oral medication; (2) pregnant and lactating women; (3) with mental illness and cognitive impairment; (4) received antiviral drugs (nirmatrelvir/ritonavir, azvudine, molnupiravir, monoclonal antibodies) in the week before admission; (5) with respiratory failure requiring mechanical ventilation, shock, ICU care required for other organ failure, or clinically predicted to have no hope of survival.

### Trial design and study protocol

**Figure 1:**
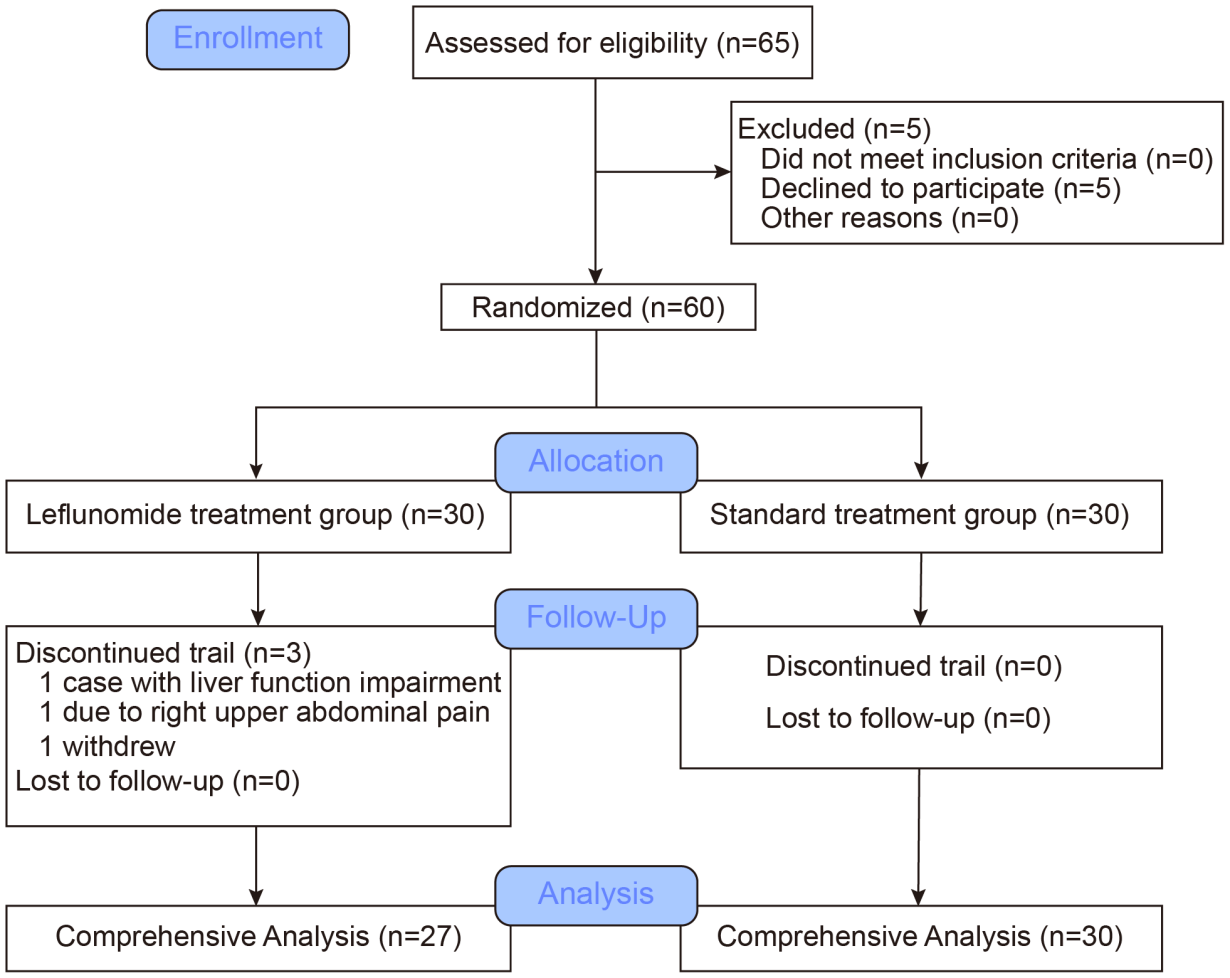
Study flow chart.

Using the random number table method, symptomatic patients with acute COVID-19 who met the inclusion/exclusion criteria were randomly assigned to either the leflunomide group (leflunomide plus symptomatic treatment) or control group (symptomatic treatment alone), as shown in Figure 1. All participants were given symptomatic treatment, e.g., antipyretics, analgesics, antiemetic or antidiarrheal medicines, traditional Chinese medicine, supportive therapy, or supplemental oxygen as necessary. Patients in the leflunomide group were additionally treated with leflunomide (50 mg orally every 12 h for three consecutive doses, then 20 mg once daily for 3 days, for a total of 5 days). Leflunomide tablets (10 mg) were provided by LongMarch-Xinkai Pharmaceutical Co., Ltd., Suzhou, China.

Before randomization, each individual completed a comprehensive questionnaire to gather data on demographics, comorbidities, symptoms and timing of the initial episode, symptom severity (Supplementary Table S1), chest imaging, blood tests, and comprehensive treatment strategy. Then, a comprehensive questionnaire containing COVID-19-related symptom scores, peripheral blood cell analysis, biochemical indicators, and chest imaging, hospitalization status, and treatment regimen were recorded by study personnel at the same time each day until the COVID-19-related symptoms resolved or day 21, whichever was earlier. Meanwhile, viral shedding time was measured using antigen detection kits or SARS-CoV-2 RNA qRT-PCR kits (GeneoDx Biotech Co, Ltd., Shanghai, China), and the time from diagnosis to two consecutive negative measurements was considered the virus clearance time.

### Outcomes

The primary efficacy endpoint was the duration from randomization grouping to sustained clinical recovery through to day 21, where clinical recovery was defined as a total score ≤ 1 for all COVID-19-related symptoms (as shown in Supplementary Table S1, higher scores indicate greater severity on a scale of 0 to 3, with a total score of 20 items ranging from 0 to 60) on two consecutive days. Secondary efficacy endpoints included the viral shedding time, fever duration, proportion of patients progressing to severe disease or requiring intensive care, peripheral blood leukocyte, death after enrollment, pneumonia, and biochemical parameters. Safety outcomes included adverse events (AEs) occurring during treatment, serious AEs (sAEs), and premature discontinuation of treatment.

### Statistical analysis

Prism 8.0 software (GraphPad Software, La Jolla, CA) was used for statistical analysis. Normality of distributions was tested with the Kolmogorov–Smirnov test. Non-normally distributed data are presented as medians [interquartile range (IQR)], and the Wilcoxon rank sum test was used to compare groups. Categorical data are expressed as cases (%), and groups were compared with the *χ*^2^ test or Fisher’s exact test. The Wilcoxon rank sum test or CMH test was used for ranked data. Median time to recovery from clinical symptoms, fever duration, and median duration of viral shedding were all estimated using the Kaplan–Meier method and compared with the log-rank test. Hazard ratios (HRs) for duration of sustained clinical recovery and their 95% confidence intervals (CIs) were estimated using Cox proportional hazards models. Participants with missing endpoint data were considered to have no clinical recovery on the same day.

## Results

### Baseline characteristics

From December 8, 2022 to December 20, 2022, 60 patients were recruited for randomized assignment and treatment to assess the efficacy of leflunomide in acute SARS-CoV-2 (Omicron strain) infection. After enrolment, 30 patients were allocated to the leflunomide group and received leflunomide combined with symptomatic treatment, while the other 30 patients received symptomatic treatment only as the control group. All participants in this study received 3 dose COVID-19 inactivated vaccine. Twenty-seven patients (90%) in the leflunomide group received all scheduled treatments, with three patients not completing the 5-day regimen, one due to severe abdominal pain after 2 days on the drug, one due to impaired liver function, and the third subject withdrew without clear reason. As shown in Table 1, there was no significant difference in age [median (IQR), 30 (26–56) vs. 32 (24.8–40.3) years; *p* = 0.462] or sex (M:F) between the two groups (16:11 vs. 14:16, *p* = 0.493). In addition, there were no significant differences between groups in first symptoms, maximum fever temperature, COVID-19 severity, or comorbidities before enrollment.

**Table 1:** Demographic and clinical characteristics of the recruited participants.

Characteristic	Leflunomide (*n* = 27)	Control (*n* = 30)	*p*-value
Age, year [M (IQR)]	30 (26–56)	32 (24.8–40.3)	0.462
Sex (M: F), *n*	16:11	14:16	0.493
First symptoms, *n* (%)			
Fever (T ≥ 37.3°C)	15 (55.6)	19 (63.3)	0.744
Max T, °C, M (IQR)	38.9 (38.5–39.2)	39 (38.6–39.5)	0.432
Cough	5 (18.5)	6 (20.0)	0.846
Headaches	2 (7.4)	4 (13.3)	0.768
Fatigue	3 (11.1)	4 (13.3)	0.882
Pharyngalgia	11 (40.7)	9 (30.0)	0.568
Myalgia	2 (7.4)	4 (13.3)	0.768
Symptom scores, scores	17 (13–24)	14 (8–24.5)	0.179
COVID-19 severity^#^, *n* (%)			0.869
Mild	23 (85.2)	24 (80.0)	
Moderate	4 (14.8)	6 (20.0)	
Comorbidity, *n* (%)			
Hypertension	3 (11.1)	2 (6.7)	0.902
Diabetes	1 (3.7)	1 (3.3)	0.519
Coronary artery disease	2 (7.4)	2 (6.7)	0.682
COPD	1 (3.7)	0 (0)	0.958
Malignancy	0 (0)	1 (3.3)	0.958
Pharmacotherapy prior to enrollment, *n* (%)			
Antipyretics and Analgesics	14 (51.9)	14 (46.7)	0.983
Antibiotics	8 (29.6)	9 (30.0)	0.795
Glucocorticoid	3 (11.1)	5 (16.7)	0.825
LianhuaQingwen capsule^*^	4 (14.8)	7 (23.3)	0.633

Leflunomide: leflunomide plus symptomatic treatment group; Control: symptomatic treatment alone.

M, median; IQR, interquartile range; COPD, chronic obstructive pulmonary disease.

# COVID-19 severity: Mild: mild symptoms (e.g., low-grade fever, mild respiratory symptoms like cough and sore throat) without pneumonia or hypoxia. Moderate: sustained high fever (>3 days) with cough, dyspnea, radiographic evidence of typical COVID-19 pneumonia, respiratory rate < 30 breaths/min, and resting oxygen saturation ≥ 94%. Severe: meets any of the following criteria: 1. Respiratory rate ≥ 30 breaths/min; 2. Resting oxygen saturation < 94% on room air; 3. PaO2/FiO2 ratio < 300 mmHg; 4. Progressive worsening of clinical symptoms with >50% increase in lung lesion progression within 24–48 h based on radiographic findings. Critical: meets any of the following conditions: 1. Respiratory failure requiring mechanical ventilation; 2. Shock development; 3. Multiple organ failure necessitating intensive care unit monitoring and treatment. COVID-19 severity based on diagnosis and treatment protocol for novel coronavirus infection (Trial version 10), 2023. https://www.gov.cn/zhengce/zhengceku/2023-01/06/content_5735343.htm.

* LianhuaQingwen capsule, a recommended Chinese traditional medicine for COVID-19.

### Laboratory examinations

As summarized in Table 2, routine blood examinations, liver and kidney function tests were performed before and after treatment in all participants. There were no significant differences in ALT, AST, urea, white blood cell and lymphocyte counts between the two groups or pre- and post-treatment. While there were differences in creatinine between the leflunomide and control groups prior to treatment [72.5 (58.3–78.7) vs. 59.5 (51.0–67.8), *p* = 0.021], all were within normal range.

**Table 2:** Laboratory results of COVID-19 participants at enrollment and after treatment^#^.

Parameter (Normal Range)	Leflunomide (*n* = 27)			Control (*n* = 30)			*P* ^a^	*P* ^b^
	Before treatment	After treatment	*p*-value	Before treatment	After treatment	*P*-value		
ALT (9–50 U/L)	23 (17.0–32.0)	27.5 (21.0–36.0)	0.180	24 (17.2–32.)	26 (21–33.5)	0.495	0.548	0.826
AST (15–40 U/L)	24.5 (19.0–31.8)	27 (22.5–36.0)	0.087	25.5 (19.0–31.5)	25.5 (19.2–34.0)	0.739	0.810	0.380
Urea (3.1–8.0 mmol/L)	4.5 (3.4–5.7)	4.8 (4.1–6.8)	0.318	4.1 (3.6–4.7)	4.6 (4.0–5.2)	0.197	0.346	0.481
Creatinine (57–97 μmol/L)	72.5 (58.3–78.7)	70.0 (60.1–77)	0.755	59.5 (51.0–67.8)	62.0 (54.3–75.8)	0.121	0.021	0.298
WBC count (3.5–9.5 × 10^9^/L)	5.6 (3.6–7.2)	5.8 (3.8–7.1)	0.971	5.2 (4.2–7.0)	5.5 (4.0–6.8)	0.915	0.850	0.963
WBC count <3.5 × 10^9^/L, *n* (%)	5 (18.5)	4 (14.8)	1.000	3 (10.0)	2 (6.7)	1.000	0.587	0.570
Lymphocyte count (1.1–3.2 × 10^9^/L)	1.3 (1.0–1.7)	1.4 (1.3–1.7)	0.529	1.3 (1.0–1.9)	1.4 (1.2–2.1)	0.220	0.996	0.536

# The data are presented as medians and interquartile ranges. *p*-values comparing cases are from Mann–Whitney U-tests. *P*^a^ and *P*^b^ indicate the statistical values comparing the before treatment or after treatment values between the leflunomide group and control group, respectively. ALT, alanine aminotransferase; AST, aspartate aminotransferase; WBC, white blood cell.

### Outcomes

**Figure 2:**
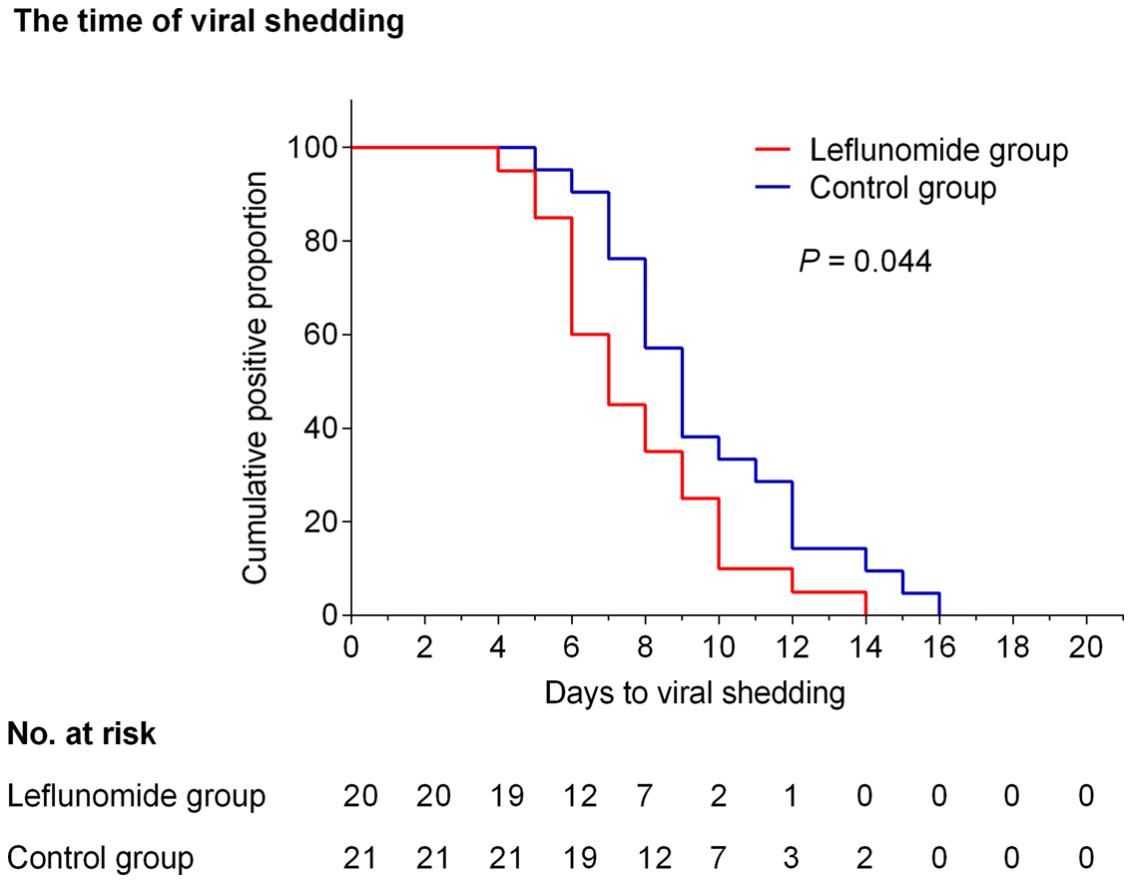
Kaplan–Meier curve showing the time of viral shedding. Compared with the control group, oral leflunomide significantly shortened the duration of viral shedding (HR, 1.718; 95% CI 1.126–4.043; *p* = 0.044). Leflunomide group: oral leflunomide plus symptomatic treatment; Control group: symptomatic treatment alone.

Clinical recovery was sustained in 27 participants in the leflunomide group and 30 participants in the control group over 21 days of follow-up, with no deaths or severe illnesses occurring. In the final analysis of this cohort, the median time from recruitment to sustained clinical recovery was 6.0 (6.0–8.0) and 7.0 (5.0–8.5) days respectively, with an HR (leflunomide versus control) of 1.329 (95% CI, 0.878 to 2.529, *p* = 0.207, Supplementary Figure S1) and no significant differences between the two groups. At follow-up, seven participants in the leflunomide group and nine in the control group were not tested for SARS-CoV-2 nucleic acid or antigen, but all had recovered from clinical symptoms. Therefore, in the statistical analysis, these data were removed and the remaining data subjected to Kaplan–Meier analysis. There was a reduction in the time of viral shedding in participants treated with leflunomide compared with those who received symptomatic treatment only [HR for negative SARS-CoV-2 nucleic acid or antigen, 1.718 (95% CI 1.126–4.043); *p* = 0.044, Figure 2]. Meanwhile, leflunomide treatment effectively reduced fever duration in patients with acute SARS-CoV-2 infection compared with controls [HR for duration of fever, 1.616 (95% CI 1.224–3.616); *p* = 0.027, Supplementary Figure S2]. There were no significant differences in hospitalization rates and incidence of pneumonia between the two groups.

With regards to safety, four patients in the leflunomide group and three in the control group reported AEs (Table 3), which was not significantly different. One participant discontinued treatment due to severe abdominal pain after 2 days of leflunomide. In contrast, no control group participant discontinued treatment due to adverse drug events. With respect to laboratory results, differences in liver and renal function between the two groups were not statistically significant (Table 2), although one patient in the leflunomide group had liver dysfunction on day two and therefore discontinued treatment (ALT 120 U/L, AST 97 U/L). No other significant laboratory abnormalities were observed.

**Table 3:** End points and adverse events in enrolled patients.

Parameter	Leflunomide (*n* = 27)	Control (*n* = 30)	*p-*value
Primary end point			
Time to sustained clinical recovery, days, [M (IQR)]	6.0 (6.0–8.0)	7.0 (5–8.5)	0.207
Hazard ratio vs. Control group, 95% CI	1.329 (0.878–2.529)	–	
Secondary end points			
Conversion to severe COVID-19 or death by day 21, *n* (%)	0 (0)	0 (0)	1.000
Duration of viral shedding, days, [M (IQR)]	7.0 (6.0–9.5)	9 (7.5–12)	0.044^*^
Duration of fever, days, [M (IQR)]	3.0 (2.0–4.0)	4.0 (3.0–6.0)	0.027^*^
Hospitalization rate, *n* (%)	8 (29.6)	13 (43.3)	0.426
Incidence of pneumonia, *n* (%)	9 (33.3)	12 (40.0)	0.806
Side effects after enrollment, *n* (%)	4 (14.8)	3 (10)	0.882
Total symptoms	3 (11.1)	3 (10)	0.768
Headaches	0 (0)	1 (3.3)	0.958
Nausea	2 (7.4)	2 (6.7)	0.682
Vomiting	1 (3.7)	0 (0)	0.958
Abdominal pain	1 (3.7)	0 (0)	0.958
Abnormal laboratory results, *n* (%)	4 (14.8)	3 (10)	0.882
Leukopenia	0 (0)	1 (3.3)	0.958
Lymphopenia	1 (3.7)	0 (0)	0.958
AST elevation	2 (7.4)	1 (3.3)	0.925
ALT elevation	4 (14.8)	1 (3.3)	0.289
Hypoalbuminemia	0 (0)	1 (3.3)	0.958

Sustained clinical recovery refers to a total score ≤ 1 for all COVID-19-related symptoms (higher scores indicate more severe disease on a scale of 0 to 3, with a total score of 20 items ranging from 0 to 60) on two consecutive days. The first day of the two consecutive-day period was considered to be the event date. Leukopenia: WBC count <4.0 × 10^9^/L; Leukopenia: Lymphocyte count <1.1 × 10^9^/L; AST elevation: AST > 40 U/L; ALT elevation: ALT >50 U/L. Hypoalbuminemia: Albumin <40 g/L. M, medians; IQR, interquartile range.

## Discussion

In this open-label RCT, we investigated the potential efficacy of leflunomide as a treatment for symptomatic mild-to-moderate SARS-CoV-2 Omicron variant infection. Fifty-seven adult participants were randomly recruited and administered leflunomide or symptomatic treatment alone within 5 days of symptom onset. The leflunomide group outperformed the control group in terms of shorter fever duration and viral shedding time.

Despite massive vaccination programs worldwide, SARS-CoV-2 continues to evolve and mutate, with the Omicron variant showing greater infectivity and immune escape compared with other strains. The China Center for Disease Control and Prevention conducted genomic analysis of specimens from SARS-CoV-2-infected patients in China from December 2022 and found a nine prevalent subtypes, with BA.5.2 and BF.7 the overwhelmingly dominant strains accounting for the national epidemic (14). However, since these variants can spread widely in a short period of time, currently recommended antiviral drugs do not sufficiently meet the needs of the general public under these circumstances. Therefore, new and effective antiviral pharmaceuticals could be helpful in reducing the medical burden.

We evaluated the effect of leflunomide as an adjuvant pharmacological therapy for patients receiving symptomatic treatment within 5 days of symptom onset and a confirmed diagnosis of COVID-19. The baseline characteristics, including initial symptoms, comorbidities, disease severity, and most baseline laboratory findings, were balanced between the two groups. Although there was no detectable difference in recovery from clinical symptoms between the leflunomide and control groups [6.0 (6.0–8.0) vs. 7.0 (5–8.5) days, *p* = 0.207], leflunomide did significantly decrease the time to virus elimination [7.0 (6.0–9.5) vs. 9 (7.5–12) days, *p* = 0.044] and fever duration [3.0 (2.0–4.0) vs. 4.0 (3.0–6.0) days, *p* = 0.027] compared with symptomatic treatment. This suggests that the immunomodulatory effect seen with leflunomide in RA and its suppression of viral replication may also be effective in COVID-19.

Within the 30 participants receiving oral leflunomide, two leflunomide recipients discontinued treatment due to abdominal pain-related AEs or abnormal liver function; however, there was no statistically significant difference in the total number of AEs between the two groups. According to the FDA, leflunomide-induced liver injury is usually mild (ALT and AST elevation ≤ twice the upper limit of normal) and it rarely induces severe liver injury (ALT and AST elevation ≥ three times the upper limit of normal); furthermore, liver dysfunction can be reversed with dose reduction or treatment cessation (11). Therefore, in the acute phase of infection, the safety of early and short-term administration of leflunomide is acceptable.

Leflunomide is an immunomodulator approved by FDA for the treatment of rheumatoid arthritis, multiple sclerosis, and lupus nephritis (15, 16). As a DHODH inhibitor, leflunomide has a broad-spectrum antiviral effect against several viruses including cytomegalovirus (17), BK viremia (18), HIV-1 (19), and Epstein–Barr virus (20). DHODH is the rate-limiting enzyme in pyrimidine biosynthesis and promotes the formation of orotate by catalyzing the oxidation of dihydroorotate, which is used as a catalytic substrate for uridine monophosphate (UMP) synthase to promote the biosynthesis of UMP (16, 21). The antiviral activity of DHODH inhibitors is also due in part to their induction of antiviral genes encoding IFN-β1 and ISG-15 (9, 22). Since the replication of RNA viruses in host cells depends on the host’s pyrimidine biosynthesis, inhibiting DHODH can effectively suppress RNA virus replication. Consistent with this, our previous studies demonstrated that leflunomide can inhibit the replication of SARS-CoV-2 *in vitro* as well as reduce the duration of viral shedding and hospitalization in a small-scale clinical study (10, 11). However, this is the first report of early leflunomide administration for improving symptoms in patients with acute stage COVID-19 after Omicron infection. At present, there is a dearth of clinical trials evaluating the efficacy of combining leflunomide with other treatment modalities for COVID-19 (23). Nonetheless, our previous research found that the adjunctive use of leflunomide in conjunction with standard-based therapy (antipyretics, analgesics, antibiotics, traditional Chinese medicine, supportive therapy, or supplemental oxygen as necessary) has demonstrated effectiveness in mitigating symptoms associated with COVID-19 (11). Moreover, we observed no discernible advantage of leflunomide in combination with IFN-α-2a on the duration of viral shedding in adult COVID-19 patients exhibiting prolonged polymerase chain reaction positivity (12). However, currently, there is a lack of research in this area, and further studies, including clinical trials, are necessary to comprehensively assess the benefits and potential risks of combining leflunomide with the current treatment strategies for COVID-19.

This trial has several limitations, not least the limited sample size and non-blinded study protocol. Furthermore, due to the poor availability of recommended anti-SARS-CoV-2 drugs, we could not compare our approach with other drugs. Additionally, the symptom assessment scales completed by some participants may have been moderately subjective, potentially biasing the results. Therefore, the results need to be validated in more heterogeneous populations with a greater variety of viral variants.

## Conclusion

In summary, our data show that leflunomide administration early in the course of acute COVID-19 Omicron variant infection can effectively reduce fever symptoms and shorten viral shedding time. However, further well-designed, multi-center, and large-sample studies are now required to confirm efficacy.

## Data availability statement

The raw data supporting the conclusions of this article will be made available by the authors, without undue reservation.

## Ethics statement

The studies involving human participants were reviewed and approved by the Ethics Committee of Renmin Hospital of Wuhan University (WDRY2021-K122) reviewed and approved the study protocol, and the study was registered with the Chinese Clinical Trial Registry (ChiCTR2100051684, registered on September 30,2021; and refreshed on March 5, 2023). Written informed consent was obtained from each participant. The patients/participants provided their written informed consent to participate in this study.

## Author contributions

ZP, ZW, YW, SZ, JZ, and HC collected the epidemiological and clinical data. ZP, ZW, and JZ were responsible for enrollment and clinical monitoring. YW, SZ, and HC were responsible for the distribution and storage of medicines. ZP and YW were responsible for statistical data. ZP and KH drafted the manuscript. KH was responsible for funding, study conception and design, and revising and submitting the final manuscript. All authors contributed to the article and approved the submitted version.

## Funding

This work was supported by the National Key Research and Development Plan for the Emergency Management of Novel Coronavirus Pneumonia (No. 2020YFC0845100) and the Science and Technology Key Project on Novel Coronavirus Pneumonia, Hubei Province, China (project number: 2020FCA002).

## Conflict of interest

The authors declare that the research was conducted in the absence of any commercial or financial relationships that could be construed as a potential conflict of interest.

## Publisher’s note

All claims expressed in this article are solely those of the authors and do not necessarily represent those of their affiliated organizations, or those of the publisher, the editors and the reviewers. Any product that may be evaluated in this article, or claim that may be made by its manufacturer, is not guaranteed or endorsed by the publisher.

## References

[1] Dejnirattisai W Huo J Zhou D Zahradník J Supasa P Liu C. SARS-CoV-2 omicron-B.1.1.529 leads to widespread escape from neutralizing antibody responses. Cells. (2022) 185:467–484.e15. doi: 10.1016/j.cell.2021.12.046, PMID: 35081335 PMC8723827

[2] Hammond J Leister-Tebbe H Gardner A Abreu P Bao W Wisemandle W. Oral Nirmatrelvir for high-risk, nonhospitalized adults with Covid-19. N Engl J Med. (2022) 386:1397–408. doi: 10.1056/NEJMoa2118542, PMID: 35172054 PMC8908851

[3] Ren Z Luo H Yu Z Song J Liang L Wang L. A randomized, open-label, controlled clinical trial of Azvudine tablets in the treatment of mild and common COVID-19, a pilot study. Adv Sci. (2020) 7:e2001435. doi: 10.1002/advs.202001435, PMID: 35403380 PMC7404576

[4] Yu B Chang J. The first Chinese oral anti-COVID-19 drug Azvudine launched. Innovation. (2022) 3:100321. doi: 10.1016/j.xinn.2022.100321, PMID: 36106026 PMC9461232

[5] Zhang JL Li YH Wang LL Liu HQ Lu SY Liu Y. Azvudine is a thymus-homing anti-SARS-CoV-2 drug effective in treating COVID-19 patients. Signal Transduct Target Ther. (2021) 6:414. doi: 10.1038/s41392-021-00835-6, PMID: 34873151 PMC8646019

[6] NH Commission (2022). Incorporate azvudine tablets into the guidelines for the diagnosis and treatment of novel coronavirus (2019-nCoV) infection (trial version 9). Available at: http://www.gov.cn/zhengce/zhengceku/2022-08/10/content_5704788.htm

[7] Cifuentes Kottkamp A De Jesus E Grande R Brown JA Jacobs AR Lim JK. Atovaquone inhibits arbovirus replication through the depletion of intracellular nucleotides. J Virol. (2019) 93:e00389-19. doi: 10.1128/jvi.00389-19, PMID: 30894466 PMC6532098

[8] Lucas-Hourani M Dauzonne D Munier-Lehmann H Khiar S Nisole S Dairou J. Original chemical series of pyrimidine biosynthesis inhibitors that boost the antiviral interferon response. Antimicrob Agents Chemother. (2017) 61:e00383-17. doi: 10.1128/aac.00383-17, PMID: 28807907 PMC5610480

[9] Luthra P Naidoo J Pietzsch CA De S Khadka S Anantpadma M. Inhibiting pyrimidine biosynthesis impairs Ebola virus replication through depletion of nucleoside pools and activation of innate immune responses. Antivir Res. (2018) 158:288–302. doi: 10.1016/j.antiviral.2018.08.012, PMID: 30144461 PMC6436837

[10] Xiong R Zhang L Li S Sun Y Ding M Wang Y. Novel and potent inhibitors targeting DHODH are broad-spectrum antivirals against RNA viruses including newly-emerged coronavirus SARS-CoV-2. Protein Cell. (2020) 11:723–39. doi: 10.1007/s13238-020-00768-w, PMID: 32754890 PMC7402641

[11] Hu K Wang M Zhao Y Zhang Y Wang T Zheng Z. A small-scale medication of leflunomide as a treatment of COVID-19 in an open-label blank-controlled clinical trial. Virol Sin. (2020) 35:725–33. doi: 10.1007/s12250-020-00258-7, PMID: 32696396 PMC7371831

[12] Wang M Zhao Y Hu W Zhao D Zhang Y Wang T. Treatment of coronavirus disease 2019 patients with prolonged postsymptomatic viral shedding with leflunomide: a single-center randomized controlled clinical trial. Clin Infect Dis. (2020) 73:e4012–9. doi: 10.1093/cid/ciaa1417, PMID: 32955081 PMC7543328

[13] Wei P. Diagnosis and treatment protocol for novel coronavirus pneumonia (trial version 7). Chin Med J. (2020) 133:1087–95. doi: 10.1097/cm9.0000000000000819, PMID: 32358325 PMC7213636

[14] Lu G Ling Y Jiang M Tan Y Wei D Jiang L. Primary assessment of the diversity of omicron sublineages and the epidemiologic features of autumn/winter 2022 COVID-19 wave in Chinese mainland. Front Med. (2023):1–10. doi: 10.1007/s11684-022-0981-7. [Epub ahead of print]. PMC10064619 37000349

[15] Wang HY Cui TG Hou FF Ni ZH Chen XM Lu FM. Induction treatment of proliferative lupus nephritis with leflunomide combined with prednisone: a prospective multi-Centre observational study. Lupus. (2008) 17:638–44. doi: 10.1177/0961203308089408, PMID: 18625636

[16] Xu Y Jiang H. Potential treatment of COVID-19 by inhibitors of human dihydroorotate dehydrogenase. Protein Cell. (2020) 11:699–702. doi: 10.1007/s13238-020-00769-9 32761523 PMC7406694

[17] Gokarn A Toshniwal A Pathak A Arora S Bonda A Punatar S. Use of leflunomide for treatment of cytomegalovirus infection in recipients of allogeneic stem cell transplant. Biol Blood Marrow Transplant. (2019) 25:1832–6. doi: 10.1016/j.bbmt.2019.04.028 31054984

[18] Nesselhauf N Strutt J Bastani B. Evaluation of leflunomide for the treatment of BK viremia and biopsy proven BK nephropathy; a single center experience. J Nephropathol. (2016) 5:34–7. doi: 10.15171/jnp.2016.06, PMID: 27047808 PMC4790185

[19] Read SW DeGrezia M Ciccone EJ DerSimonian R Higgins J Adelsberger JW. The effect of leflunomide on cycling and activation of T-cells in HIV-1-infected participants. PLoS One. (2010) 5:e11937. doi: 10.1371/journal.pone.0011937, PMID: 20689824 PMC2914784

[20] Zivadinov R Ramanathan M Hagemeier J Bergsland N Ramasamy DP Durfee J. Teriflunomide's effect on humoral response to Epstein-Barr virus and development of cortical gray matter pathology in multiple sclerosis. Mult Scler Relat Disord. (2019) 36:101388. doi: 10.1016/j.msard.2019.101388, PMID: 31525628

[21] Mei-Jiao G Shi-Fang L Yan-Yan C Jun-Jun S Yue-Feng S Ting-Ting R. Antiviral effects of selected IMPDH and DHODH inhibitors against foot and mouth disease virus. Biomed Pharmacother. (2019) 118:109305. doi: 10.1016/j.biopha.2019.109305, PMID: 31545264

[22] Lucas-Hourani M Dauzonne D Jorda P Cousin G Lupan A Helynck O. Inhibition of pyrimidine biosynthesis pathway suppresses viral growth through innate immunity. PLoS Pathog. (2013) 9:e1003678. doi: 10.1371/journal.ppat.1003678, PMID: 24098125 PMC3789760

[23] Yuan Y Jiao B Qu L Yang D Liu R. The development of COVID-19 treatment. Front Immunol. (2023) 14:1125246. doi: 10.3389/fimmu.2023.1125246, PMID: 36776881 PMC9909293

