# Supplementary material for: An open-label randomized controlled trial of leflunomide in patients with acute SARS-CoV-2 omicron variant infection
Source: Front Med (Lausanne). 2023 Jul 18;10:1218102. doi: 10.3389/fmed.2023.1218102 (PMC10392126; doi:10.3389/fmed.2023.1218102)
Supplement: Supplementary file 1 [file Table_1.docx]

| **Supplementary Table 1.** COVID-19-related symptoms and scoring | |
| --- | --- |
| Symptoms | Scoring |
| Feverish* | None = 0  Mild = 1  Moderate = 2  Severe = 3 |
| Chills |  |
| Pharyngalgia |  |
| Stuffy or runny nose |  |
| Sore |  |
| Fatigue |  |
| Headache |  |
| Dizzy |  |
| Cough |  |
| Expectoration |  |
| Dyspnea |  |
| Gasp |  |
| Pectoralgia |  |
| Palpitations |  |
| Nausea |  |
| Abdominal pain |  |
| Abdominal distension |  |
| Anorexia |  |
| Vomit | None = 0  1-2 times = l  3-4 times = 2  5 or more times = 3 |
| Diarrhea |  |
| *Note: For oral temperature, mild fever (37.3-38℃), moderate fever (38.1-39℃), severe fever (≥39.1℃). Oral temperature = tympanic temperature + 0.4℃= axillary temperature + 0.5℃[23].  Each of the 20 symptoms has a score between 0 and 3, with higher scores indicating more severe symptoms. The overall symptom score ranges from 0 to 60. | |
