# Supplementary figures and images for: An open-label randomized controlled trial of leflunomide in patients with acute SARS-CoV-2 omicron variant infection
Source: Front Med (Lausanne). 2023 Jul 18;10:1218102. doi: 10.3389/fmed.2023.1218102 (PMC10392126; doi:10.3389/fmed.2023.1218102)

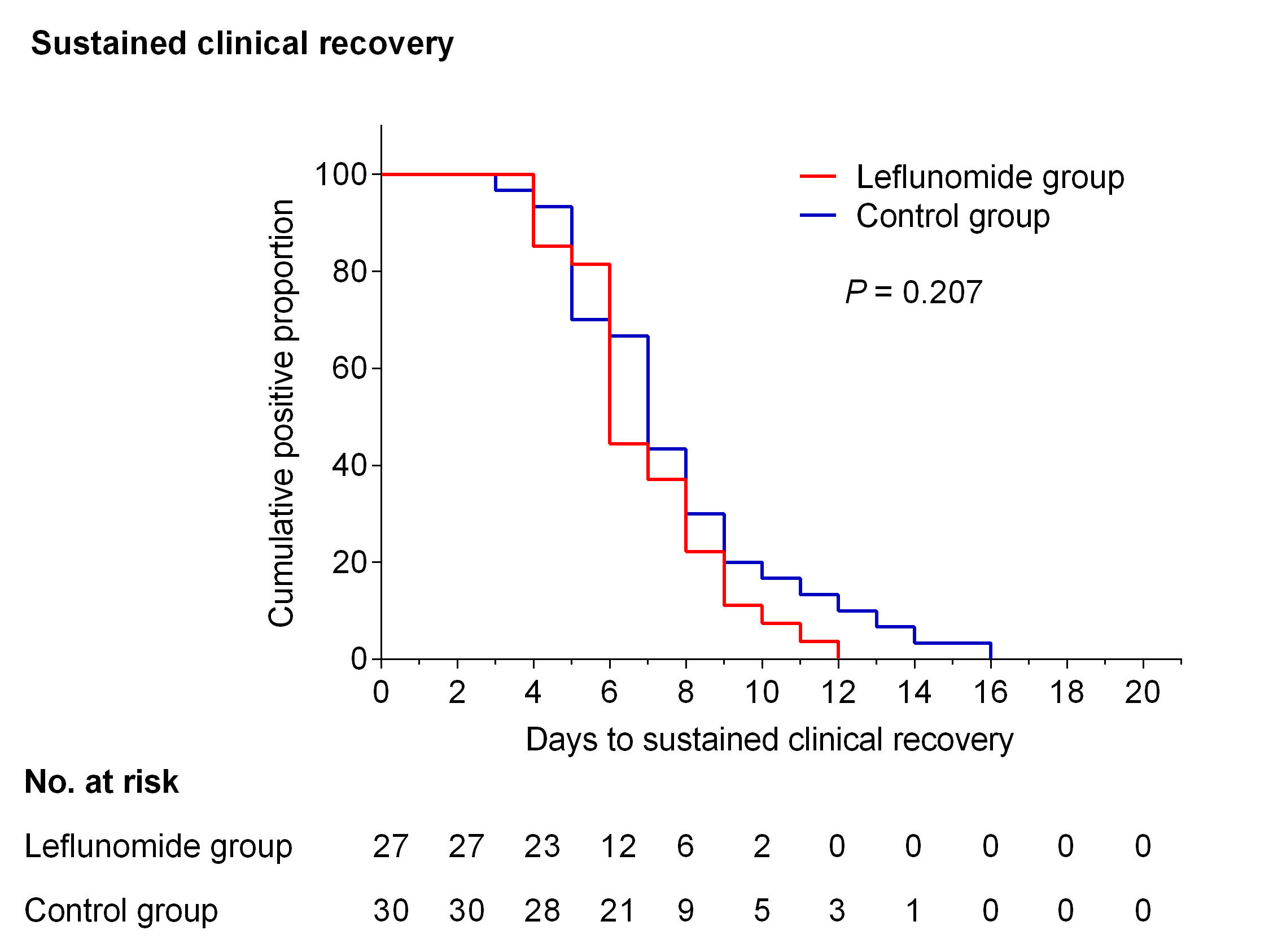

Supplement: Supplementary file 2 [file Image_1.tif]

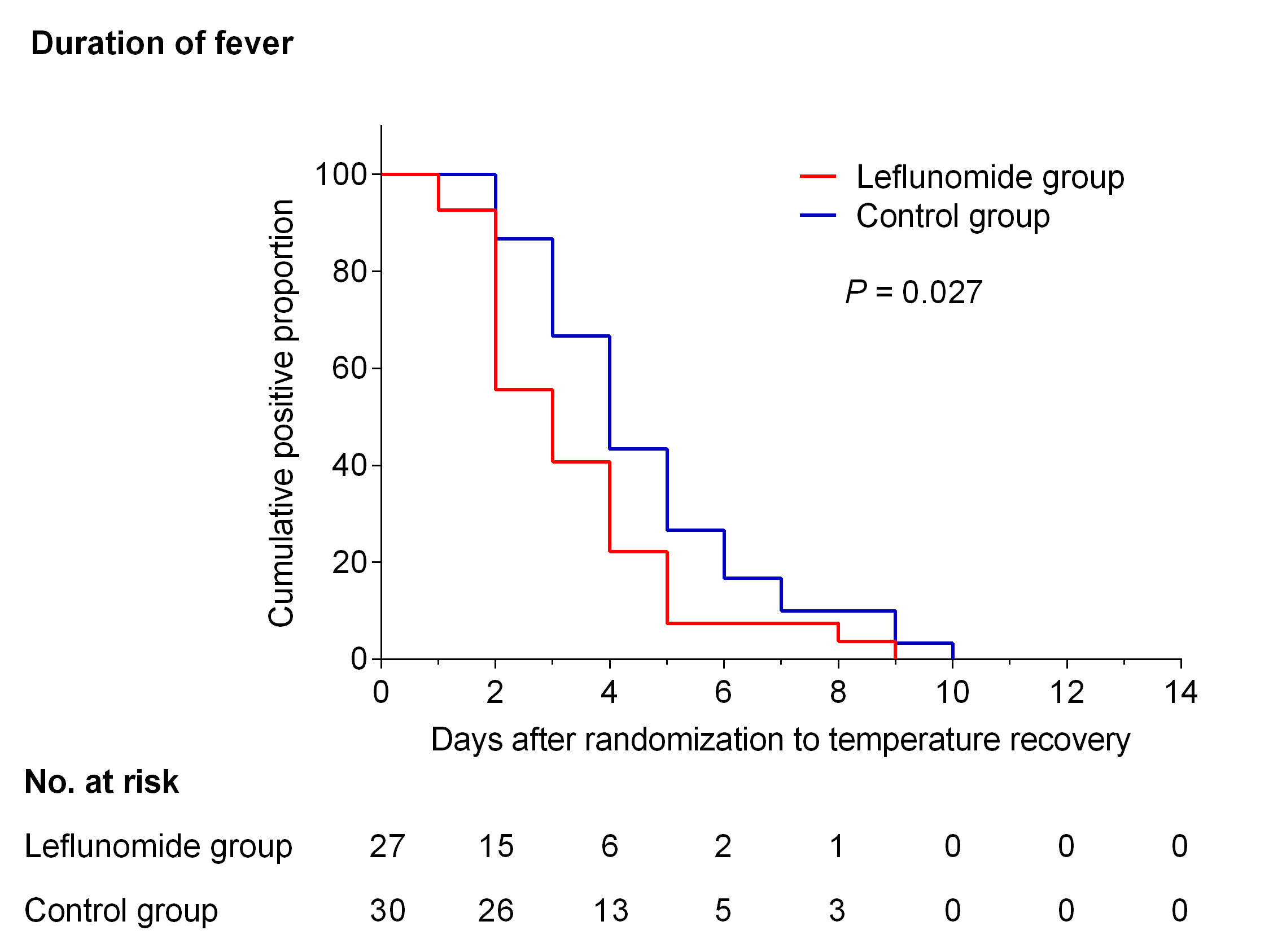

Supplement: Supplementary file 3 [file Image_2.tif]
